# The Dental Neglect Scale in adolescents

**DOI:** 10.1186/1472-6831-9-2

**Published:** 2009-01-05

**Authors:** Trilby Coolidge, Masahiro Heima, Elissa K Johnson, Philip Weinstein

**Affiliations:** 1 Dental Public Health Sciences, University of Washington, Seattle WA, USA; 2 Department of Pediatric Dentistry, School of Dental Medicine, Case Western Reserve University, Cleveland OH, USA; 3 School of Dentistry, University of Washington, Seattle WA, USA

## Abstract

**Background:**

Dental neglect has been found to be related to poor oral health, a tendency not to have had routine check-ups, and a longer period of time since the last dental appointment in samples of children and adults. The Dental Neglect Scale (DNS) has been found to be a valid measure of dental neglect in samples of children and adults, and may be valid for adolescents as well. We administered the DNS to a sample of adolescents and report on the relationships between the DNS and oral health status, whether or not the adolescent has been to the dentist recently for routine check-ups, and whether or not the adolescent currently goes to a dentist. We also report the internal and test-retest reliabilities of the DNS in this sample, as well as the results of an exploratory factor analysis.

**Methods:**

One hundred seventeen adolescents from seven youth groups in the Seattle-Tacoma metropolitan area (Washington State, U.S.) completed the DNS and indicated whether they currently go to a dentist, while parents indicated whether the adolescent had a check-up in the previous three years. Adolescents also received a dental screening. Sixty six adolescents completed the questionnaire twice. T-tests were used to compare DNS scores of adolescents who have visible caries or not, adolescents who have had a check-up in the past three years or not, and adolescents who currently go to a dentist or not. Internal reliability was measured by Cronbach's alpha, and test-rest reliability was measured by intra-class correlation. Factor analysis (Varimax rotation) was used to examine the factor structure.

**Results:**

In each comparison, significantly higher DNS scores were observed in adolescents with visible caries, who have not had a check-up in the past three years, or who do not go to a dentist (all p values < 0.05). The test-retest reliability of the DNS was high (ICC = 0.81), and its internal reliability was acceptable (Cronbach's alpha = 0.60). Factor analysis yielded two factors, characterized by home care and visiting a dentist.

**Conclusion:**

The DNS appears to operate similarly in this sample of adolescents as it has in other samples of children and adults.

## Background

Dental neglect, manifested in behaviors and/or attitudes related to the undervaluing of oral health, has been found to be a predictor of poor oral health in children and adults, measured by indices of caries, toothache, and number of teeth lost, among others [1,2]. In addition to poor oral health, dental neglect is associated with increased oral functional limitations and social and physical disabilities [3]. When dental neglect is associated with avoiding professional dental care, individuals also have lowered perceptions of their overall quality of life [4]. Thus, dental neglect in children and adults is associated with a number of negative outcomes in oral health and overall functioning.

The Dental Neglect Scale (DNS, [1]) assesses the extent to which an individual cares for his/her teeth, receives professional dental care, and believes oral health to be important. It was originally composed of 7 items and developed for parents, who were directed to rate their child's behaviors and attitude towards oral health. Children whose parents rated them as having higher dental neglect had more caries and were less likely to have gone to a dentist in the previous two years than were children whose parents rated them has having less neglect [1].

A 6-item version of the DNS has successfully been used with adults in several populations. Adults with greater neglect have more caries and fewer remaining teeth [5-7]. Their dental attendance pattern is more irregular, less likely to include recent check-ups, and marked by longer times between appointments [5,7,8]. Adults with higher DNS scores also have poorer oral health quality of life, in terms of the respondents' daily lives, social lives, and their tendency to avoid speech due to concerns about their appearance [6].

Adolescence has been identified as a time when personal oral health behaviors may be internalized and become habits, as parents become increasingly less directly involved in their children's care [9,10]. Oral care during adolescence is important for several reasons, including the eruption of permanent dentition which increases the number of tooth surfaces which may decay, and an increase in early periodontal disease [9,11]. Thus, adolescents may be at greater risk for dental disease during a developmental period when they are establishing oral care habits.

Dental avoidance is apparent in some individuals by adolescence, as youth of this age are able to influence their dental attendance [11-17]. In the UK, 48% of 16–24-year-olds go to the dentist less frequently than they did 5 years previously [16]. A second study of 14–15-year-olds in England found that about 13% of boys and 16% of girls of this age had not been to the dentist in over a year [18]. In Norway, where dental care is accessible and free for adolescents, the rates of adolescent dental avoidance may be as high as 12% or greater, and appear to increase from age 12 to 18 [17,19]. Adolescents who do not have regular dental visits have significantly more caries than their peers. For example, a study of Norwegian adolescents (for whom access was not a barrier) found that 16.4% of those who failed to visit the dentist had decayed, missing, and filled teeth (DMFT) scores more than one standard deviation above the mean, compared with 3.3% of non-avoiders [17].

Adolescents' increased autonomy may also mean that they fail to practice adequate oral home health care. For example, they may elect to consume more snacks [11]. In additions, while most adolescents report brushing their teeth twice a day, a significant number do not. Macgregor, Balding and Regis found that 28% of adolescent boys, and 14% of girls, brushed their teeth once a day, or less often (percentages computed from data presented in [20]). Taken together, these results indicate that some adolescents are neglectful of their oral care, whether by failing to visit the dentist and/or by failing to maintain good home care practices.

Since the DNS has been found to be associated with poorer oral health and irregular dental attendance in previous samples, it may be an appropriate measure for measuring dental neglect in adolescents. While the scale has been used in two samples which contained respondents aged 16 and older [6,7], to date its performance in adolescence alone has not been reported. Therefore, the primary aim of this study was to study the relationship between DNS scores, oral health status, dental attendance for routine check-up, and going to a dentist in a sample of adolescents. Inasmuch as test-retest reliability has only been reported for one sample [7], a second aim of this study was to measure the test-retest reliability in the sample of adolescents. A third aim of the study was to measure the internal reliability of the scale, as well as to carry out an exploratory factor analysis of the items in the sample of adolescents.

## Methods

This study was approved by the Human Subjects Division of the University of Washington.

### Questionnaires

Two questionnaires were used in this study. The adolescents' questionnaire contained several items, including the 6-item DNS. The adolescent answered each item on a 5-point scale, with answers ranging from "Definitely no" to "Definitely yes" for each item. Possible scores range from 6 to 30, with higher scores indicative of greater dental neglect. The adolescents' questionnaire also included an item to determine whether the adolescent had a dental office to go to as a measure of dental access, an item asking whether the adolescent currently goes to the dentist, demographic questions, and others not reported here. The second questionnaire, for parents, contained a question asking whether their adolescent had been to the dentist for a routine check-up in the past three years.

### Sample size

Using data from 16–24-year-olds in Norway [7], a subsample of 12 "high" dental neglect participants would be sufficient to find significant differences on mean DNS scores between this group and those with "moderate/low" dental neglect, at 80% power with alpha set at 0.05. Pilot work by our group in middle and high schools in a neighboring area [unpublished] found that about 82% of adolescents had a dental office to go to. Skaret et al. [17,19] found that 12% of adolescents who had a dental office to go to avoided going to the dentist. Therefore, the sample size target was set at 120–125, to be able to include approximately 100 adolescents with dental offices to go to, about 12 of whom would be likely to not go to a dentist and/or have higher DNS scores.

### Procedures

Directors of seven youth clubs in the Seattle-Tacoma metropolitan area (Washington State, U.S.) were invited to participate. The targeted clubs were located in urban, suburban, and a mixed rural/suburban area, and had youth members from a variety of racial/ethnic and socio-economic backgrounds. All seven directors agreed to participate. Youth members attended these clubs on a drop-in basis, and data collection was scheduled for the days and times when directors predicted that the maximum number of youth would be present.

Staff members at participating youth clubs distributed information about the study to parents who came to the clubs in person, and to other youth to take home to their parents. Because the youth attended on a drop-in basis, staff members were not able to inform all eligible families about the study. Parents gave written consent, and adolescents gave written assent. Parents who consented also completed the parental questionnaire and returned it with the consent forms. At the first day of data collection, dental and study personnel traveled to the location of the youth clubs. Adolescents who had completed the consent process and who were present on the day of data collection completed the questionnaire. Following this, they had a brief dental screening using light and mirror only. Calibrated dental personnel (who were not aware of the adolescents' questionnaire results) rated each tooth (except wisdom teeth, as we did not expect all adolescents to have these) for the visible presence or absence of decay, and whether or not the tooth was filled. The presence and level of decay was rated according to a modification of the WHO guide [21]. A tooth was rated as sound if there was no visible evidence of caries, as having moderate caries if there was a visible loss of tooth substance (WHO score 1 or 2), or as having severe caries if there was visibly undermined enamel (WHO score 3 or 4). Following the dental screening, the adolescent received a movie pass to thank him or her.

Study personnel returned to the youth centers weekly for the next few weeks to collect data for the second questionnaire, as not all youth were present at each visit. The adolescents completed the questionnaire a second time, and received a second movie pass. They also received a copy of their dental screening results after completing the questionnaire, so that their answers would not be influenced by learning the results of the screening. A copy of the results was sent to the parents. If the adolescent was not present during any of the days of data collection for the second questionnaire, his/her dental screening results were mailed to him/her.

### Data analyses

Data were entered into the computer and checked for accuracy. Only cases with complete data were included in the analyses. The data were analyzed with SPSS Version 14.0 (Chicago, IL). Each adolescent was given two caries ratings. First, adolescents were coded according to whether they had any visible caries or not. Second, because many adolescents had more than one carious lesion with varying degrees of severity, they were also coded according to the most serious caries rating given for any tooth. For the second coding, adolescents were coded as having no visible caries, moderate visible caries (at least one tooth with WHO score 1 or 2 but no teeth with scores 3 or 4), or severe visible caries (at least one tooth with WHO score 3 or 4). In addition to descriptive statistics, t-tests were used to compare the DNS mean scores of males and females, adolescents who had been to the dentist for a check-up in the previous three years or not, adolescents who currently go to a dentist or who do not, and adolescents who had visible caries or not. T-tests were also used to compare the six item means for adolescents who do or do not go to a dentist; to account for the multiple tests, the group-wise critical p value was 0.05, thus the critical p value for each t-test was set at 0.008 (0.05 divided by 6). A one-way ANOVA was used to examine the overall relationship between degree of dental caries (no visible caries, only moderate caries, and severe caries) and DNS scores. Post hoc tests were carried out using Tukey's Honestly Significant Differences to account for the multiple tests. Because the DNS scores were not normally distributed (most adolescents had lower DNS scores), Spearman's rho was used to analyze the relationships between age and DNS, and number of filled teeth and DNS. Intra-class correlation (two-way, mixed) was used to measure the test-retest reliability. Cronbach's alpha was used to examine the internal consistency of the DNS, and a rotated (Varimax) factor analysis was used to explore the factor structure of the items. To control for the possible effects of differences in dental access, only adolescents with a dental office to go to were included in the analyses which examined the relationships between DNS and caries, having had a check-up in the past three years, and currently going to a dentist. Data from the first administration of the DNS were used for all analyses (except for the test-retest analysis, which used data from both administrations).

## Results

### Sample demographic characteristics

One hundred and twenty-six adolescents agreed to participate. Of these, 117 (92.9%) completed the DNS on the first day of data collection and were included in the analyses. Just over half (51%) were males. Their mean age was 14.3 years (SD = 2.1, range = 12–18). The age distribution was not normal (median age = 14 years, modal age = 12 years), and 58% of participants were aged 12–14 years.

The mean DNS score for all of the adolescents was 13.2 (SD = 3.8, median = 13, range = 6–23). The mean score for males was 13.6 (SD = 4.2, median = 14, range = 6–23). For females, the mean score was 12.8 (SD = 3.3, median = 13, range = 7–23). These differences were not significant, and therefore the males and females were combined for the remaining analyses. In the combined sample, age was positively correlated with DNS scores (Spearman's rho = 0.31, p = 0.001).

### Reliability and factor analyses

The internal reliability (Cronbach's alpha) of the DNS was 0.60. The exploratory factor analysis yielded two factors with Eigen values greater than 1, explaining 54.61% of the total variance. Item loadings and total variance explained for the two factors are shown in Table 1. Four of the items (#1, 4, 5 and 6) fell on the first factor, and the remaining two items (#2 and 3) fell on the second factor. Adolescents who currently go to a dentist scored significantly lower on these two items (t = 8.76, df = 106, p < 0.001 for item #2; t = 4.48, df = 106, p < 0.001 for item #3) than adolescents who do not. There were no significant differences on the other four items.

**Table 1:** Results of Varimax Factor Analysis for Dental Neglect Scale (DNS) Items

Item	Factor 1	Factor 2
1. I keep up my home dental care.	**0.65**	0.28
2. I receive the dental care I should.	0.17	**0.84**
3. (Reversed) I need dental care, but I put it off.	0.02	**0.85**
4. I brush as well as I should.	**0.79**	-0.05
5. I control snacking between meals as well as I should.	**0.52**	0.08
6. I consider my dental health to be important.	**0.64**	0.05
Eigen value	2.03	1.25
Variance explained	29.21%	25.40%

Sixty six adolescents completed the DNS a second time. Most (60%) of the adolescents completed the second questionnaire after an interval of one week, 31% completed it after two weeks, and the remaining 9% completed it after three weeks. The test-retest reliability (intra-class correlation, two-way mixed) was 0.81 (p < 0.001).

### Validity analyses

Ninety seven (83%) of the adolescents stated that they had a dental office to go to. Almost half (47%) of them were male, and their mean age was 14.0 (SD = 2.1, median = 13, range = 12–18). Their mean DNS scores were 12.8 (SD = 3.8, median = 13, range = 6–23). There were no gender differences for DNS scores in this subsample, and thus the males and females were combined for the remaining analyses. DNS scores were positively correlated with age (r = 0.297, p = 0.003).

Of adolescents who had dental offices to go to, 21% had not been for a check-up in the past three years, 19% currently do not go to a dentist, and 31% had visible caries. Table 2 shows the descriptive statistics for the mean DNS scores for adolescents who have a dental office to go to according to whether they had been to the dentist for a check-up in the past three years, whether they currently go to a dentist, and whether they had visible caries. Adolescents who do have a dentist but who had not been to the dentist for a check-up in the past three years had significantly higher DNS scores than those who had been for a check-up (t = 2.94, df = 87, p = 0.004). Adolescents who have a dentist but who stated that they do not currently go to the dentist had significantly higher DNS scores than those who do go (t = 4.00, df = 88, p < 0.001). In addition, the DNS scores were significantly higher for those with visible caries than with those without visible caries (t = 2.17, df = 95, p = 0.032).

**Table 2:** Dental Neglect Scale (DNS) Scores by Dental Attendance and Presence and Severity of Visible Caries in Adolescents Who Have a Dentist

Had a Check-Up in the Past	Mean DNS Score	SD	Median	Range
Three Years				
Yes (n = 70)	12.1	3.6	12	6–23
No (n = 19)	15.0	4.2	15	7–23
				
Currently Goes to a Dentist				
Yes (n = 73)	12.1	3.5	12	6–21
No (n = 17)	15.9	4.0	15	11–23
				
Has Visible Caries				
No (n = 67)	12.3	3.8	12	6–23
Yes (n = 30)	14.0	3.5	14	7–23
				
Worst Visible Caries				
No Visible Caries (n = 67)	12.3	3.8	12	6–23
Moderate Caries (n = 24)	13.5	3.6	14	7–23
Severe Caries (n = 6)	16.2	2.4	15	14–20

Of the 30 adolescents with visible caries, 24 were coded with moderate caries. The remaining 6 adolescents were coded with severe caries. The descriptive statistics for the three groups (no visible caries, only moderate caries seen, severe caries seen) are shown in Table 2. The DNS means of the three groups were significantly different from one another (F = 3.66, p = 0.029). Post hoc comparisons revealed that the DNS means were not significantly different between those adolescents with no visible caries and those with moderate caries. However, those with severe caries had significantly higher DNS scores than the adolescents with no visible caries (p = 0.038).

The number of filled teeth was summed to create a measure of history of caries. Adolescents with higher DNS had significantly more filled teeth (Spearman's rho = 0.22, p = 0.03).

## Discussion

This is the first study examining the Dental Neglect Scale in adolescents. Our research design involved a sample of convenience which may limit the generalizability of our findings to adolescents in general. Nevertheless, our findings indicate that the Dental Neglect Scale appears to operate in similar ways in adolescents as it has been previously found to act in samples of parents rating their children, young adults, and adults in general.

We found that the degree of dental neglect in adolescents who have a dentist to go to was significantly related to whether they had been to the dentist for a routine check-up in the previous three years, according to parental report, as well as whether they currently go to a dentist according to their own report. In addition, adolescents with greater dental neglect were significantly more likely to have visible caries, severe caries, and more filled teeth. These three criteria – dental attendance for routine check-up, currently going to a dentist, and oral health status – have been described by previous authors as examples of criteria for the validity of the scale [1,5,7,8]. The fact that these criteria were assessed by different methods (self-report, parental report, and clinical examination) adds to the evidence of validity [22]. In addition, we found evidence for the internal and test-retest reliabilities of the DNS in our sample. Thus, it appears that the DNS has good psychometric properties when administered to adolescents.

DNS scores were significantly related to age, with older adolescents having greater levels of dental neglect. Previous studies of adults have found that the youngest adults (aged 16–24 or 18–34) had higher DNS scores than either all of the older adults, or all but the oldest group (65 and older) [6-8]. In our sample, the modal age was 12 and nearly 60% of the adolescents were aged 12 to 14. Therefore, the fact that we found a positive relationship between dental neglect and age may understate the actual extent of an increase in middle and older adolescents as a whole. The finding that older adolescents have higher dental neglect is consistent with that reported by Skaret et al. [19], who found that dental avoidance increased as adolescents age. This finding is troublesome, from a public health standpoint, as it seems to imply that increased autonomy in adolescence may be associated with poorer oral health behaviors.

The factor analysis yielded two factors, one which included the three items concerning home care plus the item assessing the respondent's overall view of the importance of dental health, and a second factor which included the item about receiving dental care as well as the item about putting off needed dental care. In our sample, dental neglect related to home care appears to capture the first factor, while neglect related to visiting the dentist appears to capture the second factor. These results are somewhat different from two of the three previously-reported analyses for adults and parents rating their children. Thomson et al.'s [1] factor analysis of the original 7-item scale yielded two factors, a general factor ("dental neglect") including all 7 items and a second factor ("avoidance of care") for the two items regarding putting off needed care. While Skaret et al. [7] reported that all 6 items in the adult DNS loaded on one factor in a sample of adults aged 16 to 79 years, he did not find evidence for a second factor. In the third previously-reported analysis, Thomson and Locker [5] stated that the single item about putting off needed dental care in the 6-item adult DNS did not load with the others in a sample of young adults. An examination of their factor loadings indicates that the item about receiving dental care loaded more highly with the second factor, characterized by the item about putting off needed dental care, than with the primary factor (loadings of 0.84 vs. 0.31). Thus, our findings are fairly similar to this previous study of young adults.

The results of our factor analysis also appear to be similar to those recently reported by Sanders, Spencer and Slade [3], who created a 10-item scale of dental neglect. In their factor analysis of responses given by adults aged 18 to 91, 5 items fell on a factor which they describe as capturing home oral self-care, while the other 5 fell on a separate factor, related to visiting a dentist. In our sample, there were significant differences between adolescents who do or do not go to a dentist only for the two items related to visiting a dentist, which comprise the second factor. Thus it appears that adolescents endorse similar attitudes with regards to home oral self-care, whether or not they go to a dentist. However, since we did not explicitly measure home health care behaviors, it would be premature to state that adolescents who do not go to the dentist actually engage in home oral self-care behaviors to the same extent as their peers who do go to the dentist.

Skaret et al. [23] used the presence of severe caries as an indicator of dental avoidance in a sample of rural adolescents, as this clinical finding appeared to indicate that an adolescent had not received dental care in some time and had not sought out dental care even though he/she was likely to be experiencing toothache or other symptoms. We found that those with no visible caries and those with moderate caries responded similarly on the DNS, in terms of mean scores. On the other hand, those with severe caries scored significantly higher than those with no visible caries. Moderate carious lesions could be expected to be seen even in adolescents who go to the dentist regularly. In addition, because such moderate lesions are relatively small and symptom-less, an adolescent may be unaware that he/she has one. On the other hand, adolescents with severe caries are likely to experience symptoms. In addition, a lesion corresponding to the WHO code of 3 or 4 has likely been developing for some time, and thus would be more likely to be seen in an individual who has not been to the dentist for a year or longer [24,25]. Thus, our results support using the construct of dental neglect for adolescents with more severe caries, but not for those with moderate caries.

There are two potential weaknesses to our study. First, as noted above we used a sample of convenience in one geographical area and thus the results may not be generalizable to all adolescents in that area or to U.S. adolescents in general. Second, for practical purposes the dental screenings in this study were carried out with light and mirror only. It is possible that some caries codings were erroneous, since the dental personnel were not able to use probing or x-rays. However, the fact that the coding scheme required visible evidence of caries means that false negatives (under-reporting) of caries were more likely than false positives. Indeed, during the calibration it was not uncommon for dental personnel to comment on the need to be "conservative" in their coding, since they could not use other measures to confirm their experience-based beliefs that the teeth were more seriously diseased than they appeared to be. Thus, a more comprehensive dental examination might have revealed stronger relationships between DNS scores and caries.

Given that our sample was small and one of convenience, it would be desirable to conduct a follow-up study with a larger, representative sample of adolescents. If the relationships that we observed between caries and DNS (as well as between number of filled teeth and DNS) are replicated, the DNS may be an adequate substitute for a clinical screening of adolescents in certain circumstances such as community-wide assessments, just as it may be in adult samples [5]. In particular, it may be useful in identifying adolescents likely to have severe caries. However, we agree with Thomson and Locker [5] that additional validity studies should be carried out to further evaluate whether the DNS could perform this function.

It would be useful to understand what factors influence adolescents to develop dentally-neglectful behaviors and attitudes. It is of interest to note that some adolescents believe that they are the ones primarily responsible for maintaining their oral health, while others indicate that others (such as parents and/or the dentist) have this responsibility [9]. Other researchers have noted that adolescent dental locus of control is related to the number of dental visits that they have [26]. Perhaps adolescents with more external locus of control are more likely to develop dental neglect.

Finally, in addition to being used to identify dentally-neglectful adolescents, it is hoped that the DNS will be a useful variable to assess change in dental neglect. To this end, future studies can also help establish whether high DNS scores drop as a result of interventions designed to reduce dental neglect.

## Conclusion

In sum, we found good evidence for the reliability and validity of the DNS in a sample of adolescents. Thus, the scale appears to perform similarly in adolescents as it has previously been found to perform in adults.

## Competing interests

The authors declare that they have no competing interests.

## Authors' contributions

TC was the primary designer of the study. PW also contributed to the design of the study. MH calibrated the dental personnel. TC, MH and EKJ collected data. EKJ entered the data into the computer. TC analyzed the data and wrote the manuscript. All authors read and approved the manuscript.

## Pre-publication history

The pre-publication history for this paper can be accessed here:


